# A Contrast Analysis of Deformation Characteristics and Critical Dynamic Stress of Natural and Fiber-Binder Reinforced Subgrade Filler after Different Freeze-Thaw Cycles

**DOI:** 10.3390/ma16041520

**Published:** 2023-02-11

**Authors:** Jiahui Wang, Yan Li, Xianzhang Ling, Ping Yang, Yingying Zhao

**Affiliations:** 1 College of Civil Engineering, Nanjing Forestry University, Nanjing 210037, China; 2 China 19th Metallurgical Group Corporation Limited, Chengdu 610031, China; 3 School of Civil Engineering, Harbin Institute of Technology, Harbin 150090, China; 4 School of Civil Engineering, Qingdao University of Technology, Qingdao 266033, China

**Keywords:** heavy-haul railway, cumulative plastic deformation, freeze–thaw cycles, critical dynamic stress

## Abstract

To investigate the dynamic stability of natural subgrade filler (NSF) and fiber-binder reinforced subgrade filler (RSF) under cyclic load after freeze–thaw (FT) cycles, a triaxial test was conducted to determine the correlation between cumulative plastic strain (CPS) and the quantity of loading cycles, as well as the evolution law of dynamic strength and critical dynamic stress (CDS) with different FT cycles. The CPS change in the NSF and RSF shows three states (stable, critical, and destructive) with increasing vibration times. However, both fillers have different failure forms, and the curve shapes of the CPS with loading cycle quantities before and after failure are also different. With the number of FT cycles increasing, the requisite dynamic stress threshold for NSF specimen failure decreases continuously. After three FT cycles, the anti-cumulative deformation ability of the NSF decreases by approximately 32%. The anti-cumulative deformation abilities of the NSF after seven and nine FT cycles, respectively, are similar. The amelioration measures could significantly enhance the FT resistance of the NSF. After zero, one, three, five, seven, and nine FT cycles, the requisite dynamic stress threshold for the RSF to reach destruction is increased 1.52, 1.89, 1.98, 2.32, 2.2, and 2.45 times, respectively, compared to that of the NSF. A mechanical model of critical dynamic stress of the NSF and RSF that considers the FT cycle was obtained using a multivariate nonlinear regression method.

## 1. Introduction

Heavy axles, extended formation lengths, and long action times are typical characteristics of heavy-haul railway trains. Long-term cyclic loading has a great impact on the heavy-haul railway subgrade [1]. In addition, in the absence of well-graded fillers and with the frequent use of poorly graded fillers based on cost considerations during subgrade construction, the CPS of subgrade fillers is more significant, which is more likely to cause subgrade diseases [2]. Consequently, examining the features of heavy-haul railway subgrade fillers’ cumulative deformation under continuous cyclic loading is crucial to ensuring the safe and effective operation of trains.

The weakening effect of FT cycles on the dynamic performance of the railway subgrade should be taken into consideration if the railway is being built in a region of seasonally frozen soil. Many academics have extensively researched how the FT cycle affects the mechanical characteristics of soil [3,4,5,6,7,8,9,10,11,12]. In the geotechnical engineering field, many reinforcement or binder materials, such as cement, fiber, fly ash, and curing agent, are often used to improve soils’ strength and stiffness characteristics [13,14,15,16,17,18,19,20,21,22,23,24,25]. Wang et al. studied the mechanical properties of nano-clay and cement composite-modified calcareous sand through triaxial unconsolidated undrained and scanning electron microscope tests, and found that nano-clay could modify the shear properties of cement calcareous sand [26]. Zhang et al. investigated the unconfined compressive strength and stiffness of soil reinforced with a combination of fibers and lime, and found that the optimum fiber content was 1% [27]. Shirmohammadi et al. [28] found significant improvement in the strength, durability, and microstructure of lime and zeolite mixed with silt subjected to FT cycles. Research on the mechanical characteristics of improved soil under the influence of FT action shows that fiber-reinforced materials could enhance the FT resistance of soil in terms of strength and stiffness [14,29,30,31,32]. Güllü & Khudir [33] investigated the unconfined compressive strength and stress–strain behavior of fiber and lime joint-reinforced soil under FT cycles. Jiang et al. [34] investigated the effects of FT cycles and fiber length on the behavioral flexibility of iron powder improved using fiber, and found that a fiber length of 12 mm had the best reinforcing effect.

In recent years, research on the application of combined fiber and curing agent improvement measures in the railway subgrade has primarily examined the static properties of the RSF, and research on dynamic characteristics is relatively sparse. Research on the CPS characteristics of fiber-modified filler under railway load after FT cycles is even rarer. Zhao et al. investigated the dynamic properties of fiber-strengthened sand and the static feature of curing agent-stabilizing filler, focusing primarily on sand with a fine grain without taking the FT cycle into consideration [35,36].

The dynamic stability of the RSF and NSF under cyclic load after FT cycles is investigated in this essay. A triaxial test was carried out to determine the correlation between the CPS and the numerous loading cycles, as well as the evolution law of dynamic strength and CDS with different FT cycles.

## 2. Laboratory Test

### 2.1. Test Apparatus

**Figure 1:**
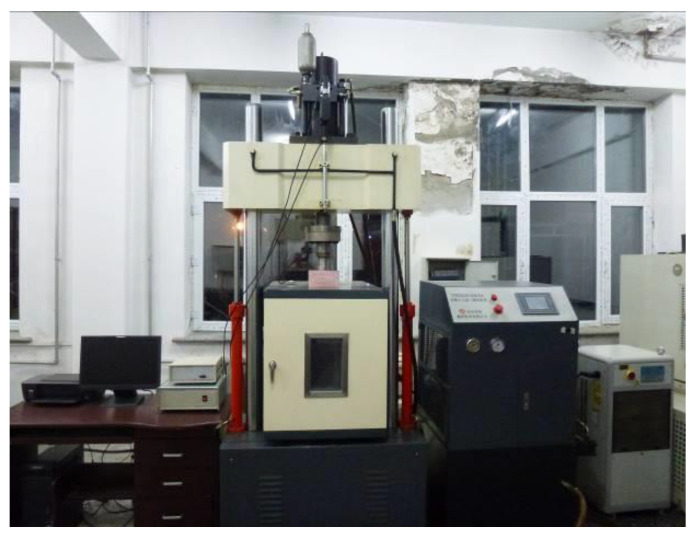
Testing apparatus (TWDSZ300).

As shown in Figure 1, tests were carried out using low-temperature dynamic triaxial testing apparatus (Suzhou TOP-TEST Instruments and Equipment Co., Ltd., Suzhou, China). The maximum axial load, axial displacement, and confining pressure could reach 300 kN, 100 mm (± 0.1%), and 20 MPa, respectively. The apparatus could control the lowest temperature of −40 ℃ with an accuracy of 0.5 ℃, and the frequency range of axial excitation was 0 to 10 Hz.

### 2.2. Test Materials and Specimens

**Figure 2:**
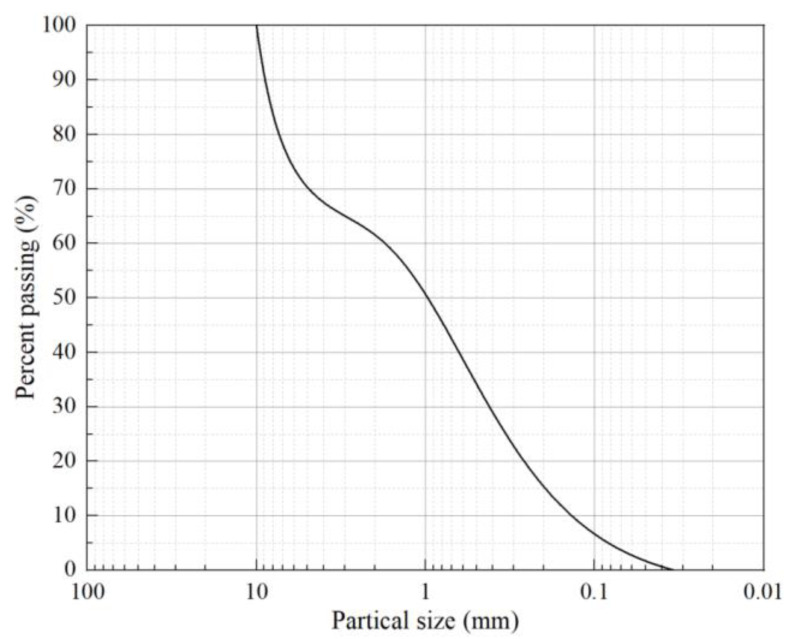
Curve representing the distribution of filler’s grain sizes.

The subgrade filler was obtained from the subgrade section of a heavy-haul railway. The filler consisted of gravel sand with soil that was poorly graded, and belongs to Group B according to the Code for the Design of the Railway Subgrade (TB10001-2016). Figure 2 is a curve representing the distribution of the filler’s grain sizes. The optimum water content and maximum dry density of NSF were determined via compaction tests as 8.2% and 2.14 g/cm^3^, respectively. Detailed information on the production enterprises for monofilament polypropylene fiber and on the research and development units of the soil curing agent are given by Wang et al. [37]. The mechanical and physical parameters of fiber and the composition of the curing agent are shown in Table 1 and Table 2, respectively. The values in Table 1 were obtained mainly according to the standard of Synthetic Fibres for Cement Concrete and Mortar (GB/T 21120-2018).

The height of dynamic the triaxial specimens was 125 mm and the diameter was 61.8 mm, and they were compacted using a three-petal mold in layers. To ensure consistency of the moisture content of the parallel specimens, all specimens were put into a water retainer for wet curing for 3 days before the FT process. Before sample preparation, the masses of dry filler, water content, fiber, and curing agent necessary for different influencing factors and improvement parameters were calculated. The NSF specimens were prepared at the optimum water content and maximum dry density, and dry soil and water were mixed evenly, and then, put into a bag to maintain freshness for 24 h before compacting. For the fiber-binder RSF specimens, to achieve the best improvement effect of the binder material, the optimal moisture content of the NSF was maintained in the following experiment of the RSF. Based on previous studies [36,39], the specimens of RSF were prepared with a maximum dry density of 2.14 g/cm^3^ with reference to that of NSF, and a water content of 12.2% (8.2% + 4%); the sum of the optimum water content and hydration reaction required water. The density of the initial RSP and NSF specimens before the freeze and thaw loads was 2.557 g/cm^3^ and 2.316 g/cm^3^. Considering the quick setting characteristics of the curing agent, the filler and fiber were combined evenly with water, and then, put into a Ziploc bag for 24 h. Subsequently, curing agent was added into the fiber-filler mixture and stirred; then, the specimens were complete with the mixture compacted in layers.

### 2.3. Freeze–Thaw Cycle and Loading Process

**Figure 3:**
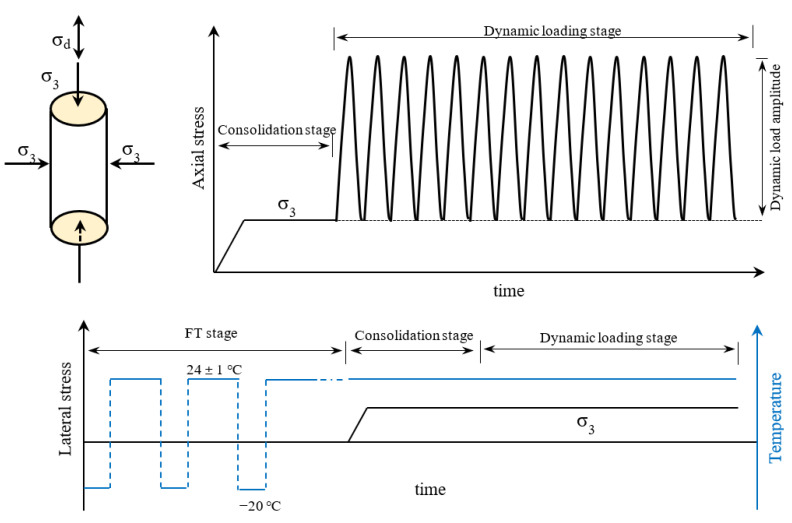
Loading process.

In the FT cycle stage, the freezing temperature was −20 ℃ like in some references [40,41]. For the FT cycle, the specimens were placed at a room temperature of 24 ± 1 ℃ for continuous thawing for 12 h after being continuously frozen for 24 h. Prior to applying mechanical stress, the FT cycle was performed in a temperature-controlled freezer using the 3D FT method without the addition of water [41]. The dynamic cyclic load applied to the railway subgrade when trains pass is a single-pulse stress with a repetitive loading process without reverse loading and unloading [42,43,44,45]. As shown in Figure 3, cyclic repetitive loading of a sine wave is used to replicate the train’s action process. The loading process is generally divided into a consolidation stage and a dynamic loading stage. In the consolidation stage, the confining pressure is constant for 2 h under drained conditions. In the dynamic loading stage, the specimen is under undrained conditions. Examining the dynamic stability of the NSF and RSF under repeated train loading, a number of loading cycles of 20,000 and axial strain of 15% were selected as the test termination standards [39,43]. As stated in the Code for Testing Dynamic Properties of Foundations (GB/T 50269-2015), a stress value corresponding to a CPS of 5% of soil samples was selected as the dynamic strength. The loading frequency selected was 2 Hz like in some other research [42,43,46,47].

### 2.4. Test Program

Numerous elements influence the subgrade’s dynamic properties under cyclic load. The influence of FT cycles was mainly considered for the dynamic characteristics of the NSF with constant moisture content, a confining pressure of 8.2%, and 100 kPa, respectively. The triaxial test device could maintain sufficient stability when the confining pressure was larger than 100 kPa. Dynamic tests of the subgrade filler under different confining pressures were conducted, and will be analyzed in another article. This paper focuses on comparative analysis of the influence of FT cycles on the dynamic characteristics of the NSF and RSF, and combined with data from some other papers [1,48,49,50], the confining pressure was determined to be 100 kPa. In addition, the influence of FT cycles was mainly considered for the dynamic characteristics of fiber-binder RSF with constant moisture content, confining pressure, fiber length, and fiber content, and the addition of binder material at 12.2%, 100 kPa, 12 mm, 0.3%, and 7%. Referring to the existing literature [34,37,39], the authors selected the respective values for fiber length and content. The fiber content and the addition of binder material were the weight ratio of the fiber and binder material to dry the subgrade filler. Table 3 and Table 4, respectively, include the detailed test design for the NSF and RSF. The number of FT cycles was determined by referring to the existing literature [31,32,34].

## 3. Results and Discussion

### 3.1. Freeze–Thaw Cycle’s Influence

**Figure 4:**
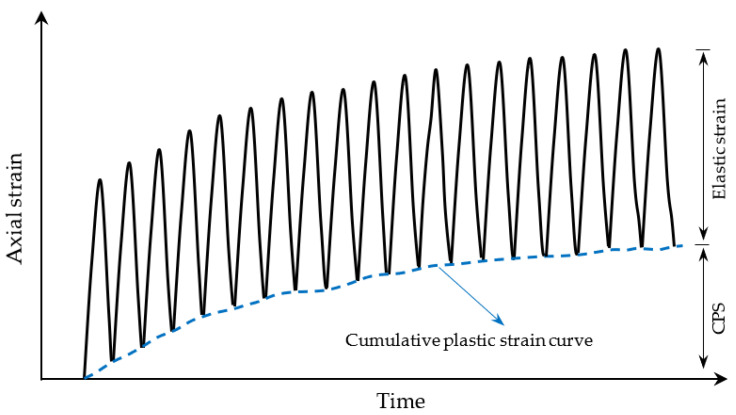
Variation in axial strain with time.

**Figure 5:**
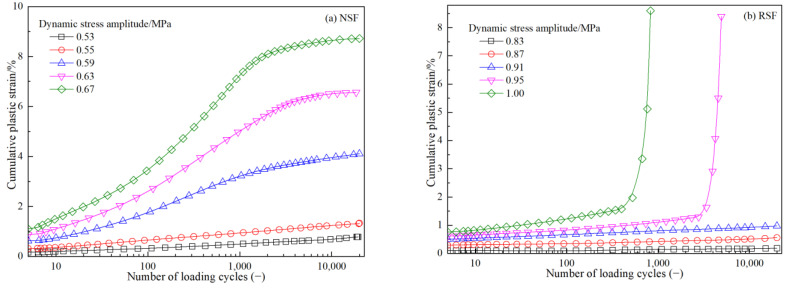

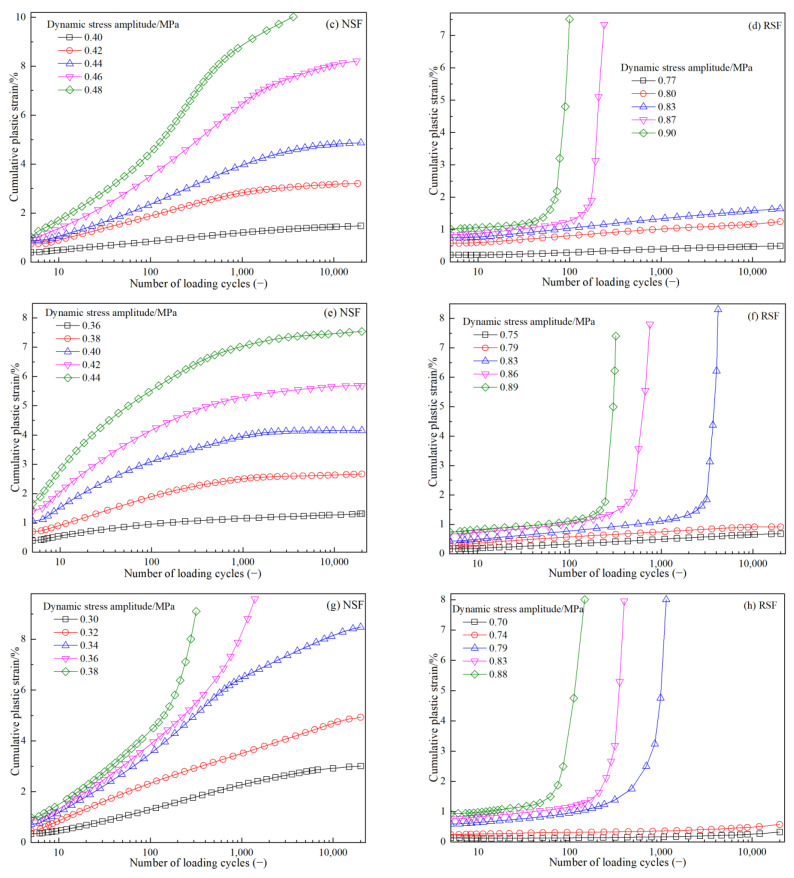

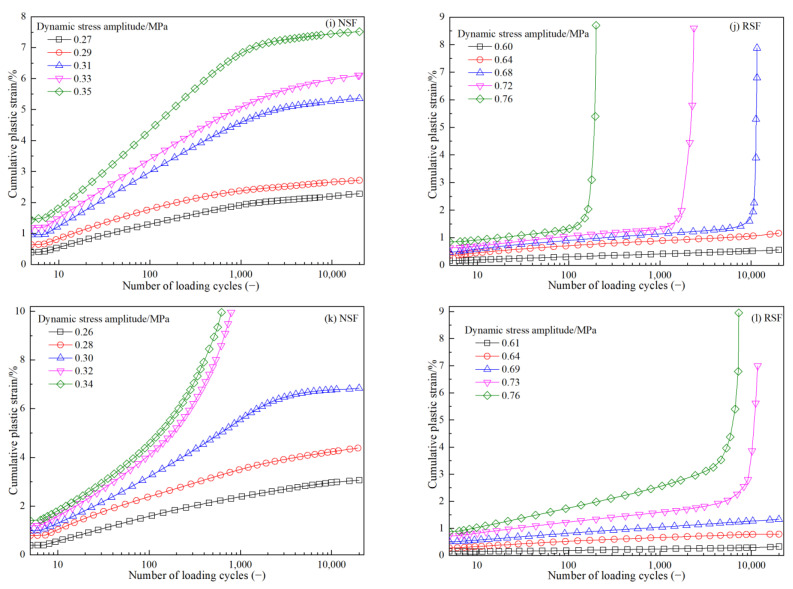
CPS and vibration number curves of subgrade filler under different FT cycles. (**a**) for NSF with FT cycle of 0, (**b**) for RSF with FT cycle of 0 (**c**) for NSF with FT cycle of 1, (**d**) for RSF with FT cycle of 1 (**e**) for NSF with FT cycle of 3, (**f**) for RSF with FT cycle of 3 (**g**) for NSF with FT cycle of 5, (**h**) for RSF with FT cycle of 5 (**i**) for NSF with FT cycle of 7, (**j**) for RSF with FT cycle of 7 (**k**) for NSF with FT cycle of 9, (**l**) for RSF with FT cycle of 9.

The CPS is the continuous accumulation of plastic strain for subgrade filler under cyclic load, as shown in Figure 4. Figure 5 shows a comparison of relationship between CPS and the vibration times of NSF and RSF under different dynamic stress amplitudes. Under various dynamic stress conditions, the CPS of NSF and RSF shows three states (stable, critical, and failure) with an increase in vibration times. However, both fillers have different failure modes, and the curves of CPS and vibration times before and after failure are also different. The natural fillings show plastic failure formation, the strain–vibration curve is “s”-shaped before failure, and the curve is “s”-shapes or a hyperbolic shape after reaching the failure standard. The RSF specimens show brittle failure, and the CPS curve in respect to vibration number is approximately linear before failure, while the curve is approximately exponential after failure with a strain turning point.

For the NSF at the same dynamic stress intensity, the cumulative strain of the specimen after no FT cycles is the smallest, and the ability to resist CPS is the largest. With an increase in FT cycles, the maximum dynamic stress amplitude of the specimen decreases gradually. After zero, one, three, five, seven, and nine FT cycles, the amplitude range of dynamic stress required for NSF specimen failure is, respectively, 0.59~0.63, 0.44~0.46, 0.40~0.42, 0.32~0.34, 0.29~0.31, and 0.28~0.30 MPa. The needed dynamic stress level for sample failure continually reduces as the number of FT cycles rises. As shown in Figure 5a,b,e,f, compared with the specimen with no FT cycles, the specimen with three FT cycles has an approximately 32% reduction in its anti-cumulative deformation ability. In Figure 5i–l, there is little difference between the anti-cumulative deformation ability of the NSF after seven and nine FT cycles, respectively. After the NSF is improved by fiber with a length of 12 mm and content of 0.3%, and by a curing agent with content of 7%, with different FT cycles, the CPS is small when the level of dynamic stress is minimal, and the sample would undergo brittle failure with high dynamic stress. The dynamic stress levels necessary for RSF specimen failure after zero, one, three, five, seven, and nine FT cycles are, respectively, 0.91~0.95 MPa, 0.83~0.87 MPa, 0.79~0.83 MPa, 0.74~0.79 MPa, 0.68~0.72 MPa, and 0.69~0.73 MPa. Compared with NSF, after zero, one, three, five, seven, and nine FT cycles, respectively, the amount of dynamic stress necessary for the sample to fail is increased about 1.52, 1.89, 1.98, 2.32, 2.2, and 2.45 times by the improvement measures. Therefore, improvements in the fiber-curing agent could significantly increase the NSF’s CPS resistance when the FT cycle and dynamic load are coupled.

**Figure 6:**
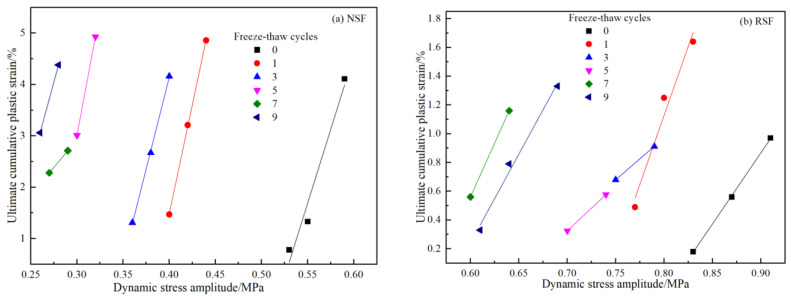
Ultimate CPS and dynamic stress amplitude curves (**a**) for NSF, (**b**) for RSF.

Figure 6a,b show the relationship between the ultimate CPS and the dynamic stress amplitude of the NSF and RSF samples under different FT cycles, with a vibration number of 20,000 and CPS less than 5%. For the NSF, with the number of FT cycles increasing, the dynamic stress amplitude needed by the specimen decreases gradually under the same CPS conditions. As shown by the interval between the curves, after one FT cycle, the amplitude of dynamic stress required by the specimen decreases the most. After five FT cycles, the amplitude of dynamic stress and the CPS of the specimen are relatively stable. The slope of the stress–strain curve increases with the increase in FT cycles the first five times, which indicates that the more FT cycles there are, the faster the CPS increases. Thus, the growth rate would slow down after five FT cycles. For the RSF, with an increase in FT cycles, the stress–strain curve swings to the left, then, shifts back to the right after seven FT cycles. Similar to the NSF specimens, the dynamic stress amplitude of the RSF specimen decreases the most under the same cumulative strain conditions after one FT cycle. The difference is that the dynamic stress amplitude of the RSF is larger than the value of the NSF, and the CPS is obviously smaller than the corresponding value of the NSF.

**Figure 7:**
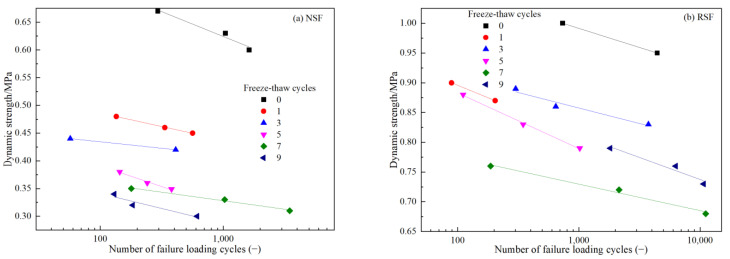
Curves of dynamic strength and failure vibration numbers (**a**) for NSF, (**b**) for RSF.

Figure 7a,b show the connection between the dynamic strength and failure vibration times of the NSF and RSF under different FT cycles, respectively, with the CPS reaching 5% and the vibration number at less than 20,000. In Figure 7a, under the same vibration times, the NSF’s dynamic strength decreases continuously with the increase in FT cycles. After one FT cycle, the sample’s dynamic strength decreases the most, and the dynamic strength tends to be steady after five FT cycles. The NSF specimens’ dynamic strength decreases with the increase in loading times, and has a logarithmic linear relationship with the failure vibration times. In Figure 7b, the dynamic strength of RSF specimens with different FT times decreases linearly with the failure vibration times. The RSF specimens’ dynamic strength first falls when subjected to the same vibration times; then, it increases as FT times rise. As shown in Figure 7b, the curve interval displays the amount of dynamic strength decrease in the RSF specimens after FT, and the strength attenuation range of the RSF specimen is the largest after one FT cycle, like that of the NSF specimen. The difference between the NSF and RSF is that the dynamic strength of the RSF specimens recovered after three and nine FT cycles. The reason is that the internal structure of the specimens, such as the filler particles, fibers, cements, moisture, and pore structure, changes in terms of arrangement and interaction, which are affected by FT cycles and dynamic loads.

### 3.2. Interfacial Mechanical Interaction

**Figure 8:**
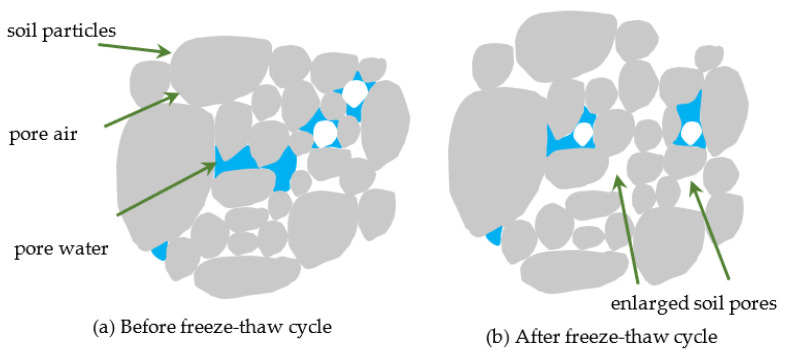
Interfacial mechanical interaction sketch of NSF.

**Figure 9:**
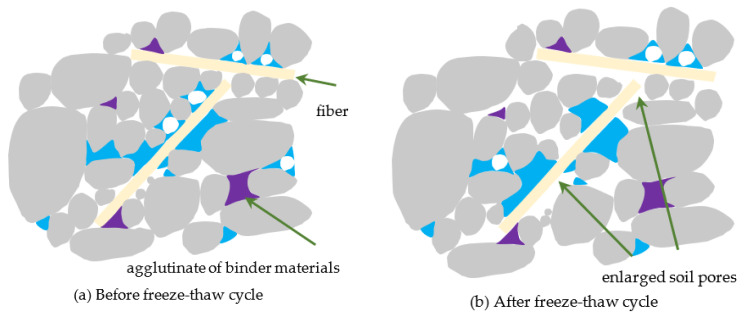
Interfacial mechanical interaction sketch of RSF.

From the comparison of the relationship between the CPS and loading times, dynamic stress amplitude, and failure strength of the NSF and RSF, it is seen that the deformation and strength characteristics of NSF under the coupling action of the FT cycle and the long-term cyclic dynamic load could be significantly improved by adding fiber and a curing agent. The mechanism of this phenomenon is analyzed from a microscopic perspective. Figure 8 and Figure 9 show an illustration of the internal mechanical interaction of the NSF and RSF before and after an FT cycle, respectively. Figure 8 shows that the voids between the soil particles of the NSF would become larger due to the phase change and migration of moisture after the FT cycle, weakening the interaction between the soil particles [51,52]. Therefore, the CPS resistance of the NSF would be weakened and the dynamic strength would attenuate under the long-term cyclic dynamic load. In Figure 9, the RSF also undergoes an attenuation phenomenon after the FT cycle. However, due to the filling of filler pores by fibers and curing agent products, fibers and soil particles interacting, as well as the cementation between curing agent products and soil particles, the weakening effect of the FT cycle is offset; thus, the RSF has better cumulative deformation resistance and strength characteristics under the coupling action of long-term cyclic dynamic load and the FT cycle. These results are consistent with past studies by Kravchenko et al. on the impact of FT cycles on fiber-reinforced clay [53].

## 4. Critical Dynamic Stress

According to a soil triaxial test under cyclic loading, a scholar named Health from the UK concluded that there were three types of curve between the CPS and the vibration times: attenuation curve, failure curve, and swing curve. The swing curve is dividing line to distinguish the attenuation curves and failure curves. The dynamic stress level equivalent to the swing curve is called the CDS, which is related to the soil type, moisture content, compaction degree, confining pressure, and FT cycles. To control the CPS in the embankment, the amplitude of the long-term cyclic dynamic stress must be less than the CDS when the railroad is running normally. The CDS’ upper and lower limits are determined from the dynamic stress of the closest attenuation and failure curves in this study. Then, the CDS’ mathematical formula, which takes the impact of FT into account, is provided, serving as reference for related engineering.

**Figure 10:**
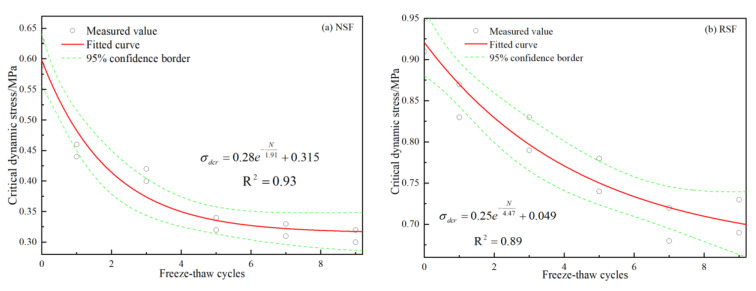
Variation in critical dynamic stress with FT cycles (**a**) for NSF, (**b**) for RSF.

Figure 10a shows a fitting curve of the CDS of the NSF depending on the number of FT cycles. The CDS of the NSF decreases with the increase in FT cycles, and the attenuation amplitude of the CDS caused by one FT cycle is the largest. After five FT cycles, the stress attenuation amplitude gradually decreases, and the CDS gradually tends to become stable, as determined in some other research on dynamic and resilient modulus changes in soil after six FT cycles [54,55]. The fitting curve of the CDS of the NSF with the FT cycles can be expressed by an exponential function, which is similar to the dynamic compressive strength change in sandstone [56]. Most of the selected test results fall within the 95% confidence interval, and the fitting degree of the curve is good. Figure 10b shows the variation in the CDS of fiber-binder RSF with the FT cycles. The variation in the CDS of the RSF with FT cycles is similar to that of the NSF, whereby the dynamic stress value decreases with the increase in FT cycles. The dynamic stress level recovers after nine FT cycles, and the specimen’s interior morphology typically exhibits stability under FT conditions. The fitting curve of the CDS and FT cycles of the RSF can be characterized by an exponential function, while the fitting degree of the NSF is better than that of the RSF. Compared with the NSF, the CDS level of the RSF is obviously improved, and is about 1.55~2.2 times that of the NSF under the same FT conditions.

Figure 10a shows the correlation between the CDS of the NSF and FT cycles. The relationship between the CDS and FT cycles was obtained from the normalized analysis, and can be expressed as follows.

(1) $$\sigma_{\mathrm{dcr}}=0.28e^{\frac{N}{1.91}}+0.315$$

Figure 10b demonstrates the relationship between the CDS of the RSF and FT cycles, whose relationship was obtained via normalization and is expressed as follows.

(2) $$\sigma_{\mathrm{dcr}}=0.25e^{\frac{N}{4.47}}+0.049$$

## 5. Conclusions

The relationships between the CPS and vibration number, the ultimate CPS and dynamic stress amplitude, the dynamic strength and failure vibration times of the NSF, and the fiber-binder RSF under the coupling action of FT cycles and long-term cyclic dynamic loading were studied through a dynamic triaxial test. The internal mechanism of the influence of FT cycles and dynamic loading on the dynamic characteristics of the NSF and RSF was comparatively analyzed, and the relationship between CDS and FT cycles under long-term dynamic load was obtained.

(1) At various dynamic stress levels, the relationships between CPS and the vibration number of the NSF and RSF show three states: stable, critical, and failure. However, the NSF specimens show plastic failure, and the curve of CPS with vibration number is mostly “s”-shaped before failure, while the curve is “s”-shape or hyperbolic after reaching the failure standard. The RSF specimens show brittle failure, and the curve of CPS with vibration number is approximately linear before failure, while the curve is approximately exponential after failure with a strain turning point.
(2) Under FT cycle conditions, the anti-CPS performance of the NSF with no FT cycles is the strongest. With the increase in FT cycles, the dynamic stress level required for NSF specimen failure decreases continuously. After three FT cycles, the anti-cumulative deformation ability of the NSF decreases by approximately 32%. There is little difference between the anti-cumulative deformation ability of the NSF after seven and nine FT cycles, respectively.
(3) Compared with the NSF, after zero, one, three, five, seven, and nine FT cycles, the fiber-binder RSF specimens’ dynamic stress levels increase approximately 1.52, 1.89, 1.98, 2.32, 2.2, and 2.45 times, respectively, before they reach failure states. These findings demonstrate that the fiber-binder reinforcing technique could significantly increase the NSF’s CPS resistance under the coupling action of FT cycle and dynamic load.
(4) Similarly to that of the NSF, after one FT cycle, the dynamic strength attenuation of the RSF sample is the largest. The difference is that the dynamic strength of the RSF would recover after three and nine FT cycles.

## Figures and Tables

**Table 1:** Physicomechanical characteristics of polypropylene fiber [37,38].

Type	Density /g·cm^−3^	Diameter/µm	Melting Point /℃	Ignition Point /℃	Elongation at Break /%	Elasticity Modulus /GPa	Tensile Strength /MPa
Fascicular monofilament	0.91	31	165~170	590	30	≥3.5	≥350

**Table 2:** Curing agent chemical makeup [37].

Composition	Silicate	Aluminate	Tetracalcium Aluminoferrite	Sulfate	Silicon Dioxide	Sulfur Aluminate
Content	14%	36%	21%	9%	3%	17%

**Table 3:** Design of dynamic triaxial testing for NSF.

Test No.	Moisture Content/%	Confining Pressure/kPa	Freeze–Thaw Cycles
DU01	8.2	100	0
DU02			1
DU03			3
DU04			5
DU05			7
DU06			9

**Table 4:** Design of dynamic triaxial testing for RSF.

Test No.	Fiber Length/mm	Fiber Content/%	Addition of Binder Material/%	Freeze–Thaw Cycles
DM01	12	0.3	7	0
DM02				1
DM03				3
DM04				5
DM05				7
DM06				9

## Data Availability

The data used to support the findings of this study are available from the corresponding author upon request.
